# Harnessing the power of proteins in modulation of miRNAs for targeting Iron deficiency Anemia: Opinion for future implications and strategies

**DOI:** 10.3389/fnut.2025.1535498

**Published:** 2025-01-27

**Authors:** Ray Wagiu Basrowi, Tonny Sundjaya, Dessy Pratiwi, Nurlinah Amalia, Yosi Yohanes Putra Tandi, Muhammad Yasir Syafa’atulloh, Garuda Nusantara Putra Utomo, Muhammad Abdir Rahman Albarok, Fahrul Nurkolis

**Affiliations:** ^1^Department of Community Medicine, Faculty of Medicine, Universitas Indonesia, Jakarta, Indonesia; ^2^Health Collaborative Center (HCC), Jakarta, Indonesia; ^3^Danone Specialized Nutrition, Jakarta, Indonesia; ^4^Department of Epidemiology, Faculty of Public Health, Universitas Indonesia, Jakarta, Indonesia; ^5^Medical Study Program, Faculty of Medicine, Brawijaya University, Malang, Indonesia; ^6^Master Program of Biomedical Science, Faculty of Medicine, Brawijaya University, Malang, Indonesia; ^7^Medical Research Center of Indonesia, Surabaya, Indonesia; ^8^Bachelor of Medicine, Faculty of Medicine, Universitas Padjadjaran, Bandung, Indonesia; ^9^Medical Study Program, Faculty of Medicine, Airlangga University, Surabaya, Indonesia

**Keywords:** miRNA, microRNA, anemia, erythropoiesis, protein, iron deficiency anemia, erythrocyte

## Abstract

Iron Deficiency Anemia (IDA) remains a pervasive global health challenge, disproportionately affecting vulnerable populations such as women and children. This review explores the cutting-edge interplay between microRNAs (miRNAs) and proteins in erythropoiesis, highlighting novel therapeutic strategies for IDA. Emerging evidence underscores the pivotal role of miRNAs—such as miR-15a, miR-24, miR-150, and miR-223—in regulating erythropoiesis, with dysregulation linked to hematologic and systemic diseases. Proteins, acting as modulators of miRNA activity, present innovative pathways for intervention by influencing erythropoiesis at multiple stages, from stem cell proliferation to red blood cell maturation. Our synthesis highlights key molecular mechanisms: miR-15a suppresses erythropoiesis by inhibiting c-Myb, miR-24 impairs heme biosynthesis through ALK4 regulation, while miR-150 and miR-223 modulate critical hematopoietic pathways affecting cell differentiation and apoptosis. These miRNA-protein interactions suggest targeted therapies such as protein-based miRNA modulators could optimize erythropoiesis, advancing IDA management. Additionally, the review emphasizes the potential of leveraging protein-miRNA interactions for precision medicine, especially in resource-limited settings where anemia’s burden is profound. By bridging current knowledge gaps, our proposed strategies offer personalized and scalable therapeutic solutions. This comprehensive perspective lays the groundwork for future interventions addressing one of the world’s most widespread public health crises.

## 1 Introduction

Anemia is characterized by hemoglobin levels falling below the threshold required to support the physiological demand for oxygen transport by circulating red blood cells. It remains a significant global health challenge. In 2021, the global prevalence of anemia across all age groups was 24.3%, equating to approximately 1.92 billion people worldwide (1). This prevalence varies across countries, with the highest rates in South Asia, Western Sub-Saharan Africa, and Central Sub-Saharan Africa (2). Anemia affects approximately one-third of the global population and is linked to increased morbidity and mortality, particularly among women and children. It contributes to adverse birth outcomes, reduced work productivity in adults, and impaired cognitive and behavioral development in children. Preschool children (PSC) and women of reproductive age (WRA) are especially vulnerable to the effects of anemia (3). Despite extensive public health efforts, the global burden of anemia has persisted, prompting a deeper exploration of its underlying biological mechanisms. While iron deficiency remains the most recognized cause, emerging research points to complex molecular processes driving impaired red blood cell production. Recent scientific advances have identified the regulatory role of microRNAs (miRNAs) in erythropoiesis, highlighting them as key molecular players in anemia pathogenesis.

Managing anemia continues to be a considerable challenge, particularly in Indonesia. Despite various interventions, such as iron and folic acid supplementation, the prevalence of anemia has not significantly declined (4). One of the key factors contributing to anemia is nutritional deficiency, particularly protein. During protein malnutrition, structural and cellular changes in the hematopoietic microenvironment contribute to bone marrow atrophy and impair hematopoietic stem cell formation, disrupting hematopoietic homeostasis and leading to anemia (5). Furthermore, animal studies have demonstrated that protein deficiency can induce anemia by inhibiting effective erythropoiesis through reduced protein synthesis in erythroid cells and decreased erythropoietin production, even when iron stores are sufficient (6).

Proteins, serving as modulators or co-factors of miRNA activity, further shape this regulatory network. Understanding these intricate miRNA-protein interactions opens new avenues for therapeutic interventions aimed at correcting dysregulated erythropoiesis. At the biomolecular level, the red blood cell formation process, or erythropoiesis, is regulated by various molecular mechanisms, including microRNA (miRNA) modulation. Notably, miRNAs such as miRNA-15a, miRNA-24, miRNA-150, and miRNA-223 play critical roles in this process. Dysregulation of these miRNAs has been linked to diseases like thalassemia, leukemia, sickle cell disease, and cancer. Moreover, miRNAs are crucial in erythropoiesis as they regulate gene expression in erythroid progenitor cells’ proliferation, differentiation, and apoptosis. For example, miRNA-15a suppresses erythropoiesis by inhibiting the expression of the MYB protein, which is essential for the kinetics of mature erythroid cells, while miRNA-24 impedes terminal differentiation by regulating the ALK4 gene (7). By shifting the focus from traditional iron supplementation to biomolecular targets such as miRNAs and their associated protein regulators, innovative strategies can be developed to combat anemia more effectively. This molecular perspective not only enhances the understanding of anemia’s root causes but also paves the way for precision medicine applications tailored to individual patient profiles.

In recent years, scientific research has increasingly focused on biomolecular aspects to understand the fundamental mechanisms behind red blood cell formation and anemia. A key area of interest is the role of proteins in this process, as proteins play a pivotal role in regulating various biochemical pathways involved in red blood cell production and maintenance. For instance, proteins such as erythropoietin promote cell survival and drive terminal erythroid maturation (8), while transcription factors like GATA1 regulate all aspects of erythroid maturation at the transcriptional and functional levels (9). However, comprehensive studies exploring the relationship between proteins and miRNAs in anemia are still lacking despite the potential of this area in developing anemia management strategies. This review aims to bridge this gap by examining the modulation of miRNAs by proteins and exploring how these interactions could offer novel therapeutic strategies to combat anemia, particularly in populations most affected by this condition.

This opinion paper uniquely explores the interplay between proteins and miRNAs in erythropoiesis, offering a novel biomolecular perspective on combating iron deficiency anemia. By integrating recent evidence and highlighting therapeutic potentials, this discussion not only advances the scientific understanding of anemia management but also paves the way for globally scalable interventions aimed at mitigating one of the most prevalent and impactful health challenges worldwide.

## 2 Search strategy and study selection criteria

To identify relevant studies, a comprehensive literature search was conducted using major scientific databases including PubMed, Scopus, Google Scholar and Web of Science. The search strategy employed combinations of key terms such as “microRNA,” “miRNA modulation,” “iron deficiency anemia,” “erythropoiesis,” and “protein regulation.” Only peer-reviewed articles published in English were included to ensure up-to-date insights.

The inclusion criteria were: (1) original research or systematic reviews focused on miRNA and protein interactions in erythropoiesis; (2) studies highlighting therapeutic applications for IDA; and (3) articles reporting molecular mechanisms with experimental or clinical evidence. Exclusion criteria included non-English publications, studies lacking relevance to IDA, and conference abstracts or editorial pieces. Titles and abstracts were screened for eligibility, followed by full-text evaluations of selected articles. Data extraction focused on molecular pathways, key findings, and potential therapeutic implications. This systematic approach ensured a robust and focused analysis of the current landscape of miRNA and protein-based interventions in IDA management.

## 3 The health beneficial of protein

### 3.1 The fundamental role of protein in anemia

Proteins are biopolymers made up of amino acids. In the human body, they serve many crucial roles, such as providing the body’s building blocks, function as hormones, enzymes, precursors of several biologically relevant molecules, and initiators of cellular death (10). One of the key roles of protein is its involvement in the development of anemia. Hemoglobin (Hb), an important protein responsible for transporting oxygen to body tissues, serves as one of the markers for diagnosing anemia in humans (11). A reduction in hemoglobin levels below 13.5 g/dl in men and 12.0 g/dl in non-pregnant women, regardless of the underlying cause, is indicative of anemia. Structural abnormalities in hemoglobin, such as those seen in conditions like thalassemia and sickle cell disorders, can lead to anemia because of premature destruction of red blood cells (12).

**Figure 1:**
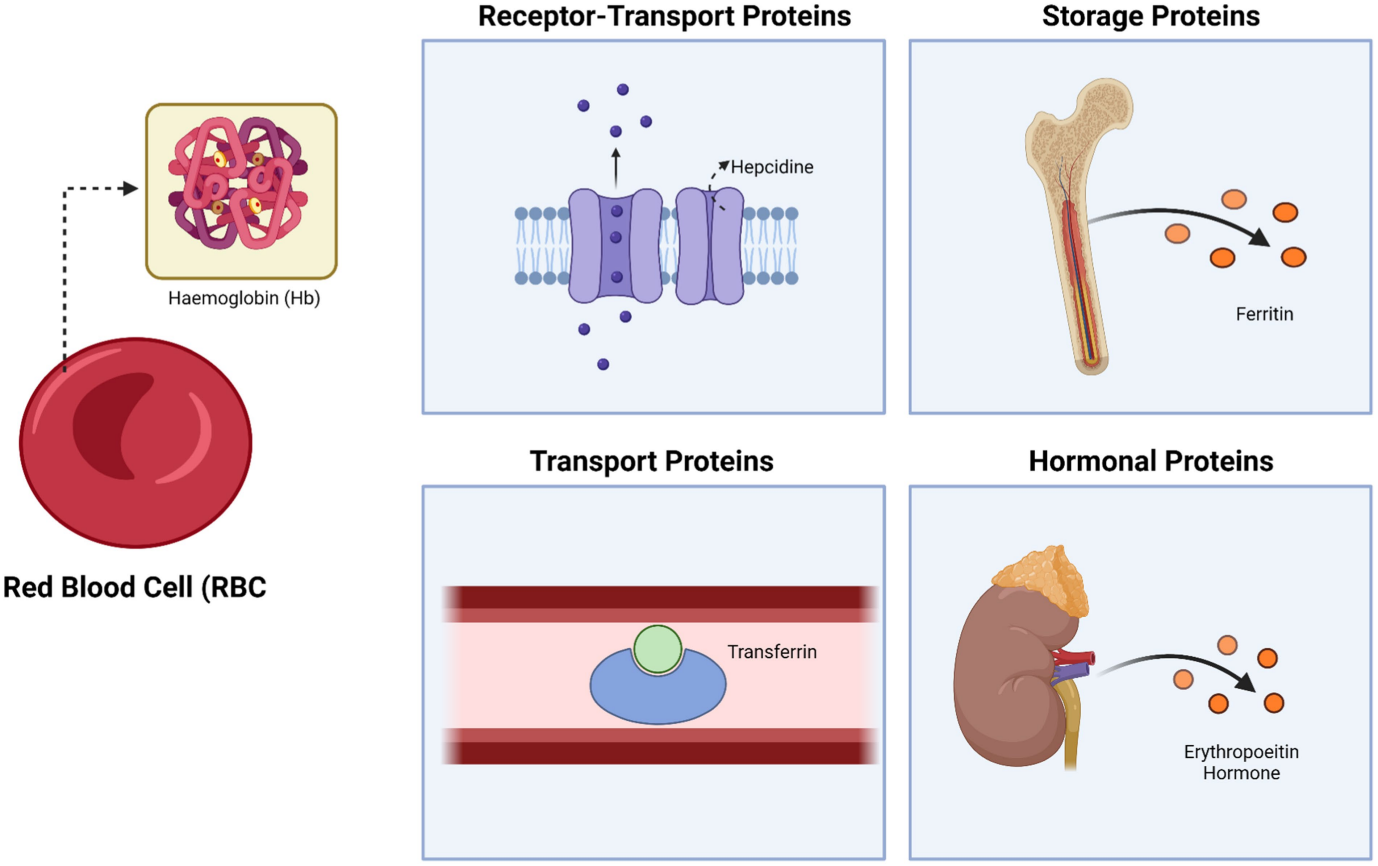
The fundamental role of proteins in anemia: protein as receptor-transport, storage, and hormonal proteins. Created with BioRender.com.

Iron is an essential component of hemoglobin and its metabolism is regulated by various proteins, including ferritin (which stores iron), transferrin (which transports iron), and hepcidin (a negative regulator of iron absorption and recycling), along with other proteins that modulate iron metabolism at different physiological levels (13, 14). Disruptions in iron metabolism can result in iron deficiency anemia (IDA), the most common form of anemia worldwide (15). The involvement of proteins in anemia is particularly evident in anemia of inflammation (AI), also called anemia of chronic disease (ACD), the second most prevalent form of anemia globally (16). Inflammatory cytokines, key proteins in this process, contribute to a reduced lifespan of red blood cells, likely by activating macrophages, while also disrupting erythropoietin (EPO) production and function, and inhibiting the proliferation and differentiation of normal erythroid progenitor cells (3). Erythropoietin (EPO) is a glycoprotein hormone synthesized by the kidneys that is essential for stimulating red blood cell production in response to low partial pressure of oxygen (pO_2_), making it important in the context of anemia. A reduction in erythropoietin levels, commonly seen in individuals with chronic kidney disease, can also lead to the development of anemia (17). Figure 1 shows the involving of protein in anemia.

Protein deficiencies disrupt erythropoietic pathways by impairing key molecular processes essential for red blood cell formation (18). Specifically, inadequate protein intake compromises the synthesis of hemoglobin, the main oxygen-carrying protein in erythrocytes, and reduces the availability of erythropoietin, a hormone critical for stimulating erythroid progenitor proliferation (19). Furthermore, protein-related transcription factors like GATA1 are essential for the differentiation of erythroid cells, while transporter proteins such as transferrin and ferritin regulate iron homeostasis required for effective erythropoiesis (20). These disruptions collectively impair red blood cell production, leading to various anemia-related conditions.

### 3.2 Differences in protein types for Anemia management

Anemia has numerous etiologies which fundamentally revolve around two core issues: inadequate production of red blood cells or hemoglobin (supply) and excessive demand for them (either physiological or pathological). The management strategy for anemia focuses on addressing the specific etiology in each patient, with the goal of achieving a balance between the two (3). In cases of iron deficiency, the most common cause of anemia, therapy focuses on restoring normal iron levels in the body, through pharmacological iron supplementation (oral or IV, if necessary) (Table 1) (21). In more complex cases, such as anemia due to chronic diseases like cancer, management options are more varied, including vitamin B12, folic acid, erythropoiesis-stimulating agents, and even blood transfusions, taking into account the appropriateness of each option, as well as their respective risks and benefits. While it is important to manage anemia as an individual disease case, this disease can cause significant burden throughout the world. Thus, WHO has formulated key strategies for global anemia prevention and control. These strategies require collaboration from various sectors, including the nutrient and food sector which encompass the provision, utilization, and education about healthy and nutrient-rich foods. The goal is enhancing the intake of essential micronutrients, particularly iron, folate, vitamin B12, vitamin A, and riboflavin, along with other micronutrients, that can be achieved through dietary diversification, food fortification, and supplementation strategies (22).

**Table 1:** How diet influences biomolecular aspects of iron deficiency anemia (IDA)?

Biomolecules	Role in iron deficiency anemia (IDA)
Iron	Absorption and Transport: Absorbed in the duodenum as heme (from animal products) and non-heme iron (from plants). Heme iron is more readily absorbed. Incorporation in Hemoglobin: Essential for hemoglobin synthesis, which carries oxygen in red blood cells. Low iron leads to microcytic anemia. Storage: Stored as ferritin in liver, spleen, and bone marrow, providing a reserve for low dietary intake periods.
Protein	Iron Transport and Storage: Proteins such as transferrin and ferritin transport and store iron for hemoglobin production. Hemoglobin and Myoglobin Production: Hemoglobin is a protein, and amino acids from dietary protein are essential for its synthesis. Enzymatic Functions: Proteins involved in the electron transport chain help in cellular energy production, important in IDA to prevent fatigue.
Interactions and dietary synergy	Enhancers of Iron Absorption: Certain proteins (like from meat) enhance non-heme iron absorption. Vitamin C also boosts non-heme iron absorption. Inhibitors of Iron Absorption: Phytates, polyphenols, and calcium can hinder absorption. Balanced intake is key to ensuring maximum absorption.
Dietary sources and recommendations	Iron-Rich Foods: Red meat, poultry, fish (heme iron), and fortified cereals, lentils, spinach (non-heme iron). Protein Sources: Eggs, lean meat, legumes, nuts, and dairy for necessary amino acids supporting hemoglobin synthesis and iron utilization.

The consumption of nutritious food should not only meet daily requirements but also provide high-quality nutrients (Table 1). One of the most important components is protein, particularly for obtaining various essential amino acids (23). There are numerous types of protein sources, generally classified as animal-based and plant-based proteins. Animal-based proteins tend to contain complete proteins and essential amino acids, which are well absorbed by the digestive system (24). High consumption of animal-based protein plays a role in anemia management due to its positive effects on iron status in the body, including blood hemoglobin levels. On the other hand, plant-based proteins typically lack complete essential amino acids in one type source and are less efficiently absorbed due to their fibrous structure, which is more difficult to digest. Therefore, individuals who follow a plant-based protein diet require a more diverse range of sources and larger quantities to meet their nutritional needs (25).

Several micronutrients required for normal erythropoiesis, such as iron, folic acid (Vitamin B9), and Vitamin B12, can be obtained from various protein sources, with their content differing across these sources. Animal-based protein sources, such as meat from four-legged animals and two-legged animals, contain good amounts of iron, particularly in the form of heme iron, which is readily absorbed by the body. Fish meat is also rich in iron, and a diet high in such sources can fully meet the daily dietary iron requirements. Plant-based protein sources (beans, nuts, peas, legumes, etc.) tend to contain non-heme iron, which has lower bioavailability. Moreover, these sources often contain iron absorption inhibitors, such as phytates (26). Folic acid, a vital micronutrient, is primarily found in animal-based protein sources like liver and kidney. In contrast, among plant-based foods, it is most abundant in leafy green vegetables, which are not typically recognized as significant sources of protein (27). Lastly, in the case of vitamin B12, there are no plant-based sources. This vitamin can be absorbed by the digestive system after being separated from the protein in meat. It is abundant in red meat (beef, lamb), all types of fish, and to a lesser extent in poultry products (28).

## 4 The role of miRNA in biomolecular process

The single-stranded structure of short nonprotein coding RNA or microRNA (miRNA), transcribed from DNA sequences, plays an essential role in gene expression. The miRNA interacts with messenger RNA (mRNA) to regulate transcription through the coding sequence as an independent gene promoter, producing a pattern that alters overall cellular processes. In this way, intracellular and extracellular miRNA act as messengers to cells, organs and systems that could alter the physiological and pathological homeostasis (29). A possible pathway to alter metabolism or paradoxically reprogramming the metabolism involves directly or indirectly diverse mechanisms, such as targeting key enzymes, transporters, transcription factors, and signalling pathways (30).

However, in order to interact with distant cells/organs, miRNA should remain stable and be able to interact and modulate transporters, enzymes and receptors. Exosomes or extracellular vesicles are mediators protecting miRNA stability and integrity. The miRNA exerts an influence on the modulation and inhibition of the inflammatory state, may result in alterations to physiological conditions in hematoimmunological processes. Furthermore the miRNA influences the modulation and inhibition of the inflammatory state (i.e., polarization of macrophages to two phenotypes M1 and M2), signalling pathway (NF-κB, TLR, STAT-3), and regulates the function of receptor cells (closely related to hypoxia and inflammation) (31). The hitchhiking of miRNA affects the motility of the transporter (vesicle), thereby enabling it to be exploited in an inactive form for the purpose of transporting from the post-synaptic (32).

**Figure 2:**
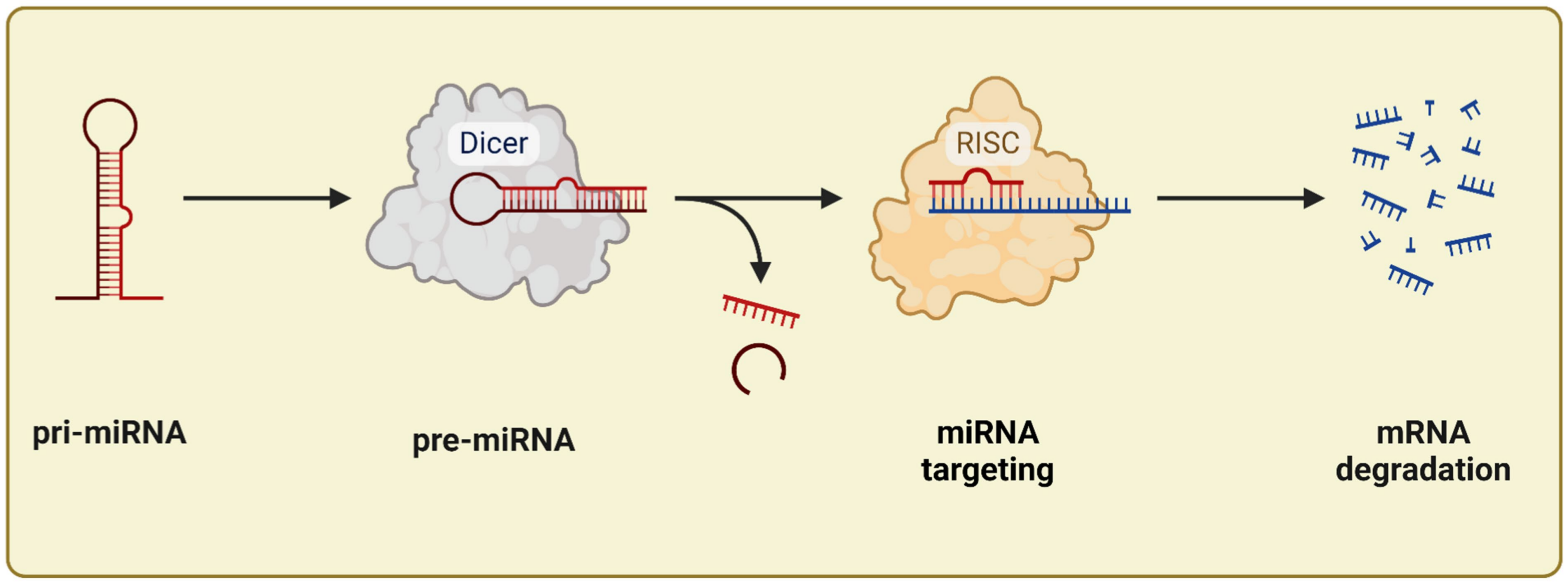
miRNA processing: start from pri-miRNA until mRNA degradation. Created with BioRender.com.

MiRNA genes are transcribed by RNA polymerase II, producing a primary transcript known as pri-miRNA. These pri-miRNAs are long sequences that contain multiple miRNA segments, which fold into characteristic hairpin structures. Within the nucleus, the pri-miRNA is processed by the Drosha enzyme, in collaboration with the Dgcr8 protein, resulting in a double-stranded precursor miRNA (pre-miRNA) approximately 70 nucleotides in length. This pre-miRNA is then exported to the cytoplasm through the XPO5-GTP complex. Once in the cytoplasm, the enzyme Dicer, assisted by the cofactor TRBP, further processes the pre-miRNA into a miRNA duplex, which is about 21–24 nucleotides long. The miRNA duplex is subsequently incorporated into the RNA-induced silencing complex (RISC), where one strand is selected to become the mature miRNA, typically around 22 nucleotides in length. This mature miRNA contains a critical “seed region” of approximately 7 nucleotides at the 5′-end, essential for recognizing target mRNAs. The miRNA binds to partially complementary sites, usually located in the 3′ untranslated regions (3’UTRs) of target mRNAs, leading to either mRNA degradation or suppression of its translation. Figure 2 illustrates the miRNA processing pathway (33).

## 5 Proteins targeting miRNA modulation in red blood cell formation

The clinical evidence of targeting miRNA in anemia for the past 10 years is summarized in Table 2. Proteins influence miRNA biogenesis, stability, and functionality through various mechanisms (34), including transcriptional regulation of miRNA-encoding genes, post-transcriptional processing by key enzymes such as Drosha and Dicer, modulation of miRNA maturation through co-factors, and interactions with RNA-binding proteins that can alter miRNA stability, localization, and target recognition (35, 36). Table 2 presents a summary of several studies conducted over the past 10 years on related to miRNA in erythropoiesis. The majority of several studies demonstrate that various types of miRNAs influence anemia through erythropoiesis. Erythropoiesis is part of hematopoiesis that plays a crucial part in erythrocyte equilibrium to maintain the physiological term. The origin of erythrocyte or red blood cell (RBC) results from the differentiation of hematopoietic stem cells (HSC) into multi-potent progenitor cells (MPP) and identified erythroid lineage called burst-forming unit erythroid (BFU-E). The BFU-E proliferates into several types of cells, such as proerythroblast. Below the regulation of erythropoietin hormone (Epo), the proerythroblast is amplified into a reticulocyte with erythrocyte as the final form of erythropoiesis. Several miRs contribute significantly to erythropoiesis, such as miR-15a, miR-24, miR-150, and miR-223. However, our tabulated studies also report several alteration targets, such as c-Myb, FOXP-1, and LMO2. Relative of several miRs (miR-15a, miR-24, miR-223) deregulated the erythropoiesis that can be found in several diseases such as thalassemia, leukemia, sickle cell disease, and cancer. The impaired erythropoiesis causes the common phenomenon of megakaryocytic cells that, in some cases, could alleviate the severity of the disease or be implicated with disease progressivity that leads to anemia condition.

**Table 2:** Summarize of included studies.

No	Author, Year	miRNA	Biological effects	Disease related of anemia	Erythropoiesis function
1	(7, 37, 38)	miR-15a	↑ suppress the expression of c-Myb blocks cell cycle progression (tumor suppressor of CLL) inhibits erythropoiesis (differentiation of erythroid and myeloid) ↑ HbF expression ameliorates the severity of sickle cell disease and β-thalassemia ↓ enhances expression of IL-7 receptor (IL7R) blocks the B-cell maturation accumulation of B1 and T cells development of CLL	Sickle Cell Disease, β-thalassemia, CLL	Deregulates (suppresses) the erythropoiesis of erythroid and myeloid (Proliferation Stage)
2	(39–43)	miR-24	↑ delays the activin pathway attenuates the erythroid differentiation reduces the formation of CFU-E and BFU-E reduces angiogenesis and prevents the migration of cancer inhibits heme biosynthesis accumulation of succinate transition defect blocks the heme synthesis ↓ induces the activin A pathway enhances EV-mediated B cell increases the survival rate of B cell acute lymphoblastic leukemia enhances terminal erythroid differentiation (erythropoiesis) induces B cell enhances the IgA secretion prevents infection	Thalassemia, cancer	Deregulates (suppresses) the erythropoiesis of progenitor proliferation and differentiation of BFU-E and CFU-E (Proliferation and Differentiation Stage)
3	(44–47)	miR-150	↑ related to the increased proliferation of megakaryocytic cells (c-Myb) inhibits early development of B cells increase platelet formation promote caspase-3 activation leads to apoptosis in pathologic cells (B cell lymphoma) ↓ related to deletion of c-Myb (terminal erythropoiesis) B1 cell proliferation IgM ↑ enhances humoral immune response interacts with FOXP1 restrict the activity of caspase-3 reduces apoptosis and influences the cell growth B cell lymphoma	B cell lymphoma, hepatocellular carcinoma	Stimulates the differentiation of MEP into BFU-E (Differentiation Stage)
4	(48–50)	miR-221/222	↑ reduces the erythropoiesis (granulopoiesis) by suppressing the activity of HSC reduces the chemosensitivity of cisplatin therapy in cancer (leukemia) ↓ Increases FOS expression drives the proliferation, differentiation, and maturation process	Cancer (Leukemia)	Suppress the terminal phase of erythropoiesis (Differentiation and Maturation stage)
5	(51)	miR-223	↑ impairs the LMO2 expression blocks the erythropoiesis process inhibits the erythroid differentiation increase the development of megakaryocytic cell increase platelet formation ↓ stimulates the LMO2 expression induces the erythroid development inhibits the E-cadherin expression induces tumor proliferation and migration induces angiogenesis increases the cancer survivability	Β-thalassemia, lymphoblastic leukemia, cancer	Regulates the erythropoiesis (HSC, erythroid cells, and granulocyte-monocyte development) (Proliferation stage)

BFU-E, burst-forming unit-erythroid; CFU-E, colony-forming unit-erythroid; CLL, chronic lymphocytic leukemia; EV, extracellular vesicle; FOS, Fos proto-oncogene; FOXP1, forkhead box P1; HbF, Fetal Hemoglobin; HSC, hematopoietic stem cell; IgA, immunoglobulin A; IL, interleukin; JUN, Jun proto-oncogene; LMO2, LIM domain only 2 MEP, megakaryocyte-erythroid progenitor.

## 6 Future directions of using protein in anemia

Previous research has identified the potential of miRNA targeting in the management of anemia, but the number of studies in this field remains limited. Moreover, variations in the markers used to assess reductions in disease incidence across studies complicate the ability to detect significant effects of miRNA specifically on anemia. Despite consistent findings over the past decade showing a strong correlation between miRNA and hematological processes such as erythropoiesis, the efficacy and efficiency of miRNA in modulating anemia-specific outcomes require further investigation.

This article underscores the need for additional clinical research to explore the connection between miRNA and anemia, particularly in identifying specific miRNAs involved in this condition. Although this paper provides indirect evidence of miRNA’s involvement in anemia through its regulation of erythropoiesis, future studies should aim to clarify its role in anemic patients, ultimately enhancing therapeutic efficacy.

### 6.1 Future directions

Advancing research into miRNA-protein interactions for IDA treatment necessitates a multidisciplinary approach involving molecular biology, bioinformatics, and clinical sciences. Proposed methodologies include:

1. Preclinical studies: conduct *in vitro* studies using erythroid progenitor cell lines to explore the effects of specific miRNA inhibitors or protein modulators. CRISPR-Cas9 technology could be employed to knock out target miRNAs, enabling precise mechanistic evaluations.
2. Animal models: utilize transgenic mouse models with altered expression of relevant miRNAs and proteins to assess phenotypic changes in erythropoiesis. Longitudinal studies could measure red blood cell counts, hemoglobin levels, and survival outcomes.
3. Omics integration: employ multi-omics approaches, including transcriptomics, proteomics, and metabolomics, to uncover new molecular targets and validate miRNA-protein interaction networks.
4. Clinical trial designs:
  - Phase I/II trials: design early-phase clinical trials focusing on safety, dosage optimization, and proof-of-concept efficacy of miRNA-targeting therapeutics.
  - Randomized controlled trials (RCTs): conduct RCTs comparing standard iron supplementation with novel protein-based miRNA modulators.
  - Biomarker-driven studies: incorporate predictive biomarkers such as circulating miRNA signatures to stratify patient populations and personalize treatments.
5. Implementation science: evaluate the scalability and cost-effectiveness of miRNA-based therapies in low-resource settings. This could involve partnership models with global health organizations.

By integrating these strategies, future research can accelerate the development of transformative treatments for IDA, bridging the gap between molecular discoveries and clinical applications.
